# Mucormycotic osteomyelitis of maxilla post-COVID patient: a case report

**DOI:** 10.11604/pamj.2021.39.275.30480

**Published:** 2021-08-27

**Authors:** Deeplata Mendhe, Pratibha Wankhede, Mayur Wanjari, Sagar Alwadkar

**Affiliations:** 1 Department of Community Health Nursing, Smt. Radhikabai Meghe Memorial College of Nursing, Datta Meghe Institute of Medical Sciences, Sawangi (M), Wardha, Maharashtra, India

**Keywords:** Fungal osteomyelitis, seldom, diabetes mellitus, paranasal sinuses, case report

## Abstract

Fungal osteomyelitis is a life-threatening and seldom seen opportunistic infection. It is commonly an affectation of the nose and paranasal sinuses within the orofacial region. It is an aggressive infection that needs to be addressed promptly to prevent fatal consequences. The mode of infection is via the inhalation route and infection begins initially in the nose and paranasal sinuses with subsequent invasion into the vascular tissue, eventually leading to thrombosis and necrosis of nearby hard and soft tissues. Here, we report a case of a 31-year-old male who presented with pain over the upper jaw that was sudden in onset, continuous, dull aching, radiating towards forehead and neck of the left side, aggravates on mastication and relives on its own. He had a history of uncontrolled diabetes mellitus. On further investigation, using diagnostic and Interventional aids, a final diagnosis of mucormycotic osteomyelitis of the maxilla was made.

## Introduction

Mucormycosis is the common name given to several different diseases caused by fungi of the order mucorales [1]. The patient generally has uncontrolled diabetes mellitus and is acidotic, has a haematological malignant disease like leukemia, or is receiving immunosuppressive therapy [2,3]. Usually, mucormycosis presents as an acute infection and manifests in rhino cerebral, pulmonary, gastrointestinal, cutaneous or disseminated forms [4], rarely affecting otherwise healthy people [5]. The infection begins in the upper turbinate or paranasal sinuses [6,7], or less commonly in the palate or pharynx. The most common presentation in the head and neck region is maxillary and orbital cellulitis in a person with inadequately controlled diabetes mellitus [7,8]. Since mucormycosis occurs infrequently, it may pose a diagnostic and therapeutic dilemma for those who are not familiar with its clinical presentations.

**Epidemiology:** according to published literature, mortality rates of mucormycosis ranges from 10% to 100% depending on the site of infection and underlying predisposing factors [9]. Maxillary sinus mucormycosis have a poor prognosis and high mortality rate (46%) [10]. Aspergillosis has a mortality rate of 30% with a poor prognosis in pediatric cases (with a mortality rate as high as 85%) [11]. Early diagnosis and immediate intervention are essential for such patients. Treatment modality includes control of the underlying risk factors, antifungal therapy, surgical debridement, supportive therapy and surgical or prosthetic rehabilitation (RECONSTRUCTION) is very important because of the restoration of quality of life to the premorbid state.

## Patient and observation

**Patient information:** a 31-year-old male patient, who was apparently alright 15 days back when he started experiencing pain over upper jaw which was sudden onset, continues, dull aching, radiating towards forehead and neck of the left side, aggravates on mastication and relieve on its own. His medical history revealed that he was a known type II diabetes mellitus patient. History of pus discharge intraorally from 21 regions and nasal pus discharge in the past 15 days approximately.

**Clinical findings:** on clinical extraoral examination, facial asymmetry due to presence of diffuse swelling over the right malar region, extending anterior-posterior ala of the right nostril to 2 cm posterior to a line perpendicular to lateral canthus of right eye superior to inferior right lower eye lied to the level of the ala of the nose. The size was 6 x 4 cm and the shape roughly oval with the surface of smooth and diffuse margins. On palpation, tenderness was present with restricted mouth opening. On clinical intraoral examination, mouth opening was 20 mm. teeth present 11-18, 21-28, 31-38, 41-48. Diffuse gingival swelling is seen in the upper right maxillary alveolar region extending from anterior to posterior 13 to 14 region. Superior to the inferior crest of the alveolar ridge to the depth of the sulcus. The shape was roughly oval and the color is reddish-pink. Multiple draining sinuses present with upper right maxillary.

**Diagnostic assessment:** the patient was hospitalized and planned for surgical debridement of the maxilla, maxillary sinus and ethmoid sinus of both sides of the affected region under general anesthesia. Before the intervention, routine biochemical, serological, and hematological examinations were done. All test reports appeared within normal limits except significantly elevated fasting blood sugar (FBS) and post prandial blood sugar (PPBS) levels (244 and 436 mg/dl, respectively). These were monitored and controlled in consultation with the endocrinologist by initiating an appropriate insulin regime.

**Therapeutic intervention:** as said earlier, fungal osteomyelitis is more invasive than bacterial, if not diagnosed and treated earlier. The treatment given to our patient is as follows: debridement of the maxilla, maxillary sinus and ethmoid sinus of both side of the affected region under general anesthesia; antifungal medication: AMPHOMUL (amphotericin B emulsion) 1 gm/kg/day x 10 days; ceftriaxone 1 gm IV BD x 7 days; amikacin 500 mg IV BD x 5 days.

**Informed consent:** the authors certify that they have obtained all appropriate patient consent forms. In the form, the patient(s) has/have given his/her/their consent for his/her/their images and other clinical information to be reported in the journal. The patients understand that their names and initials will not be published and due efforts will be made to conceal their identity, but anonymity cannot be guaranteed.

## Discussion

Mucormycosis is a rare opportunistic fungal infection caused by a saprophytic fungus (phycomycetes). The early clinical features include perinasal paraesthesia, periorbital cellulitis, rhinorrhoea, nasal crusting with stuffiness and epistaxis with or without complaining of fever, arthralgia and weight loss which is followed by eschar formation and necrosis of the nano-facial region. Strawberry gingivitis is one of the clinical features seen. Severe infection may cause life-threatening conditions like cavernous sinus thrombosis, thrombosis of a carotid artery or internal jugular vein and death [12]. Contiguous spread of infection from surrounding soft tissue and bones due to hematogenous seeding or direct inoculation of microbes into bone results in the disease origin. In all these conditions, the vascular supply is decreased, thereby predisposing the infection. Entry of microbes into cancellous bone causes the compression of blood vessels preceded by the inflammation and edema of marrow. Severe compression of vascularity leads to ischemia and necrosis of bone. Immobile and stagnant blood leads to nidus for the development of infection. Osteomyelitis is commonly seen in males (80.36%) than in females (19.64%), with a peak incidence in 30-39 years of age [13].

Osteomyelitis involving the facial bones is rare and involvement of the maxilla is less common than that of the mandible due to high vascularity. Even though broad-spectrum antibiotics decreased the prevalence of the disease, it remains a challenging entity in developing countries and low socioeconomic groups. Osteomyelitis occurring due to fungal infection is rare and occurs in an indolent manner [13].

Medical treatment is not alone effective because of poor drug concentration and availability to the infection site due to thrombosis of the vascular system. Treatment consists of correction of underline systemic abnormality, for example, control of diabetic state, stoppage or modification of immunosuppressant or corticosteroids are essential [14].

The treatment approach includes antifungal therapy combined with surgical intervention (sequestrectomy and debridement) and adjunctive therapy. The choice of antifungal drug is AMPHOMUL (amphotericin B emulsion) therapy (1.0-1.5 mg/kg/day) [3]. Posaconazole 400 mg is to be taken twice daily [15].

## Conclusion

Immunocompromised patients are vulnerable to fatal fungal infections involving bones and soft tissues of the oral cavity. Such infections always create a dilemma for the clinicians to reach a definitive diagnosis due to the paucity of occurrence and reporting. Early diagnosis and treatment can save such consequences. The possibility of such kind of fungal osteomyelitis should be taken into account as a differential diagnosis for similar clinical situations.
